# Factors leading to cesarean section delivery at Felegehiwot referral hospital, Northwest Ethiopia: a retrospective record review

**DOI:** 10.1186/s12978-015-0114-8

**Published:** 2016-01-20

**Authors:** Fantu Eyowas Abebe, Abebaw Worku Gebeyehu, Ashebir Negasi Kidane, Gizached Aynalem Eyassu

**Affiliations:** 1 Strengthening Human Resource for Health Project, iNGO, P.O. Box, 1566 Bahir Dar, Ethiopia; 2 Institute of Public Health, University of Gondar, Gondar, Ethiopia; 3 Maternal and Child Survival Program, iNGO, Addis Ababa, Ethiopia

**Keywords:** Cesarean section, Retrospective record review, Ethiopia

## Abstract

**Background:**

Cesarean section is the commonest obstetric operative procedure worldwide. When used appropriately cesarean sections can improve infant and/or maternal outcomes. However, when used inappropriately the potential harm may exceed the potential benefit of cesarean section. Appreciating the limited information in this area the current study assessed the rate and factors associated with cesarean section in Felegehiwot referral hospital, Bahir Dar, northwest Ethiopia.

**Method:**

The study was a retrospective analysis of eligible patient records that included 2967 pregnant women who had underwent either cesarean or vaginal delivery from July 1, 2012 to June 31, 2013. The data were double entered to EPI-INFO 3.5.2 and analyzed with SPSS. Binary logistic regression model was fitted to identify independent factors associated with cesarean section.

**Result:**

The proportion of women who underwent cesarean section in this study was 25.4 %. Obstructed labor (30.7 %), fetal distress (15.9 %) and abnormal presentation (13.4 %) were the major obstetric indications for cesarean section. The odd of undergoing cesarean section was higher among mothers in rural residence (AOR = 1.63, 95 % CI: 1.21, 2.20), mothers reported to have pregnancy risk factors (AOR = 2.31, 95 % CI: 1.74, 3.07) and lower among mothers in age category of 15–19 (AOR = 0.63, 95 % CI: 0.43, 0.93).

**Conclusion:**

Obstetric factors occurring around birth, including obstructed labor and fetal distress were the main reasons leading to Cesarean Section rather than background characteristics assumed to be a risk. The results imply that there is a need for timely and accurate screening of women during obstetric care and, decision to perform cesarean section should be based on clear, compelling and well-supported justifications.

## Background

Cesarean section is the commonest obstetric operative procedure worldwide [1, 2]. When used appropriately C-sections can improve infant and/or maternal outcomes. However, when used inappropriately the potential harm may exceed the potential benefit of C-section. C-sections cost more than vaginal births and can result in increased risk to mother and baby [2, 3]. There is a growing concern that Cesarean rates have been rising for all women in the world regardless of medical condition, age, race, or gestational age. International concern over such increases have prompted the World Health Organization to suggest that CS rates should not exceed 15 % [4], with some evidence indicating caesarean section rates above 15 % are not associated with additional reduction in maternal and neonatal mortality and morbidity [5, 6].

Modern obstetrics practice for medical, social, economic and legal reasons have witnessed an increase in the primary cesarean section rates everywhere [7–16]. While the Cesarean Section rate ranges between 12 and 86 % across studies done in developed countries [6, 7, 10, 17] and the rate in developing countries vary between 2 and 39 % [2, 4, 6, 12, 18, 19].

Caesarean section delivery is increasing in Ethiopia [20], indicative of access to obstetric care service in the country. In the urbanized region of the country, the rate ranges from 8 to 37 % [20, 21]. The increment in caesarean section delivery rate in major urban cities is a cause of concern as it surpasses the WHO threshold of 15 %. However, according to the Ethiopian demographic and health survey 2011 report, only 2 % of the women had undergone caesarean section [22].

Many factors have been claimed to attribute for increased cesarean section rate across the world. While some literatures [3, 4, 6, 14] reported Premature Rupture of the amniotic fluid Membrane (PROM), Cephalic Pelvic Disproportion (CPD), fetal distress, multiple pregnancy and breech presentation as factors associated with increased rate of caesarean section. Some others revealed that it is associated with place of health seeking (private with public) and maternal preferences [9, 20, 23, 24].

Few other studies also demonstrated the relationship between cesarean section and maternal age [9, 24, 25]. Even some other studies find out that, birth weight, parity, maternal height and history of antenatal care visit (ANC) to be factors associated with cesarean section [10, 17].

Ante Partum hemorrhage (APH), multiple pregnancy, cord prolapse, mothers HIV infection condition and having previous history of cesarean section were also found to be factors leading to increased cesarean section rate [12, 18, 19]. The improved safety of surgical and anesthetic skills in modern obstetrics and mothers positive attitudes towards CS among staff and patients could also be the other factors that contribute for increased rate of cesarean section.

It has been shown that a significant number of obstetricians would agree to perform an elective CS without an obstetrical indication upon maternal request [9, 24, 26]. Currently there is much debate as to whether this surgical procedure should be performed for women without clear clinically acceptable indications [1, 6, 7]. Even, in Ethiopia, perhaps in the region little information is available with regard to the magnitude and factors associated with rate of C-section in hospitals. Thus, this study intended to assess the magnitude and factors associated with cesarean section in Felegehiwot Referral Hospital, Amara region, Northwest Ethiopia.

## Methods

### Study setting and design

The study was a retrospective analysis of eligible and complete client records that included 2967 pregnant women who had undergone either cesarean or vaginal delivery from July 1, 2012 to June 31, 2013 in Felegehiwot Referral Hospital, Amhara region, Northwest Ethiopia. This hospital is one of the five busiest referral hospitals in the region. It is proximal to serve about 5,000,000 people including pregnant women where majority of them are usually referred from neighboring zonal hospitals, health centers, health posts and private health facilities.

### Data collection and analysis

Pre-tested questioner was used to collect mothers’ information including age, parity, gestational age, antenatal care, stage of labor at admission, fetal condition at admission, reason for admission prior to intervention, onset of labor, spontaneous or induced, oxytocin infusions, instrumentation and reason for referral before admission to the hospital.

### Inclusion and exclusion criteria

A total of 3460 women were registered for maternity care, including abortion care over the year. We identified and reviewed 3063 eligible maternal charts. Of which, 60 charts were incomplete and were excluded from the study. Furthermore, 36 charts of the women who had uterine rupture were also excluded and the final sample size became 2967.

The completeness and consistency of the data was checked, cleaned and double entered to EPI-INFO software version 3.5.2 and analyzed by SPSS software version 16. Binary logistic regression model was applied to handle potential confounding variables and to identify independent factors associated with cesarean section. In order to avoid collinearity between different factors, two models were fitted independently. The first model considered more of background characteristics (more distal factors) whereas the second model focused on immediate causes of CS. Significance was taken at *P* value of < 0.05. Model fitness was checked using Hosmer and Lemeshow goodness of fit test.

### Ethical consideration

Ethical approval to conduct the study was obtained from Amhara Regional Health Bureau research ethics review committee. Communication with the hospital administration made through formal letter obtained from the regional health bureau. The data obtained from the hospital was kept confidential.

## Result

Among the 2967 eligible mothers, 723 (25.3 %) had CS delivery. The majority 653 (90.3 %) of these women had emergency CS and referred cases were responsible for the higher (79 %) proportion of emergency CS in this study. Women who were referred from other facilities constitute close to 73 % of the total study participants.

Eight nine mothers had previous CS delivery. Among these women, 30 (37.8 %) had attempted Vaginal Birth After CS (VBAC) and only 19 (63.3 %) of them had successful vaginal delivery.

Among the 271women who had induction, 32 (11.8 %) of them ended up with CS delivery. During the last pregnancy, 2321(77.3 %) of the mothers had ANC visits, of whom, 1465 (63.1 %) of the women had four or more ANC visits.

Of the total 723 CS deliveries, 364 (50.3 %) were made by general anesthesia and the remaining were spinal anesthesia. Nine mothers were reportedly died during/following CS delivery and related to the use of general anesthesia. Respiratory failure was responsible for the majority 4(44.4 %) of maternal deaths. Two women died due to hemorrhagic shock and 2 of them died due to disseminated intravascular coagulation and the remaining one was due to aspiration pneumonia. Forty-seven women (6.5 %) had unjustified CS for a dead fetus.

The detail analysis of vaginal delivery showed that there were 1855 (82.7 %) spontaneous vaginal deliveries, 349 (15.6 %) assisted vaginal/instrumental deliveries including the four assisted vaginal birth after CS, 25 (1.1 %) destructive deliveries and 15 (0.66 %) spontaneous vaginal births after previous CS.

In this study, 269 (9.06 %) newborns were stillbirths. The still birth rate for CS (excluding 36 cases due to uterine rupture) and vaginal delivery was 6.5 and 9.9 % respectively. On the other hand, ten of the 13 immediate newborn deaths were incriminated to CS delivery and the majority (80 %) of these immediate newborn deaths was related to the use of general anesthesia. However, the overall perinatal mortality rate in the reference hospital was 10.5 % (8.4 % for CS and 11.1 % for vaginal delivery).

True labor, leakage of liquor, preeclampsia, vaginal bleeding and postdate were the common causes of admission for both vaginal and CS delivery in this referral hospital (Table 1).

**Table 1:** Reasons for admission of pregnant women in Felegehiwot referral hospital, Bahir Dar, Ethiopia 2013

Reasons for admission	Route of delivery	
	All forms of vaginal delivery	CS
True signs of labor	1746 (77.8 %)	501(69.3 %)
Leakage of liquor	214(9.7 %)	58(8.0 %)
Preeclampsia/eclampsia	72(3.2 %)	44(6.1 %)
Absent fetal movement	62(2.8 %)	9(1.2 %)
Post date	53(2.4 %)	40(5.5 %)
Vaginal bleeding	52(2.3 %)	46(6.4 %)
Bad obstetric history	24(1.1 %)	17(2.4 %)
Retained second twin	18(0.8 %)	3(0.4 %)
Previous CS	0 (0 %)	5(0.7 %)
Total	2244 (100 %)	723(100 %)

Quite a significant number of mothers have had preventable complications and most of the complications were happened during or following vaginal delivery (Table 2).

**Table 2:** Intrapartum and postpartum maternal complications observed among women enrolled for the study, Felegehiwot referral hospital, Bahir Dar, Ethiopia 2013

Complications	Route of delivery		Total
	Vaginal delivery	CS delivery	
1st degree perineal tear	74(36.6)	-	74
2nd degree perineal tear	66(32.6)	-	66
Hemorrhage	35(17.4)	20(27.0)	55
Preeclampsia/Eclampsia	17(8.5)	14(18.9)	31
Wound infection	-	23(31.1)	23
Sepsis	8(3.9)	8(10.8)	16
Maternal death	0	9(12.2)	9
Vesico-vaginal fistula	2(1.0)	-	2
Total	202(100 %)	74 (100 %)	276

**Fig. 1:**
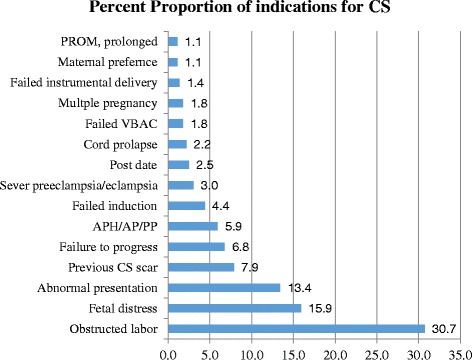
Percent proportion of obstetric indications for CS delivery, FHRH, Amhara, Ethiopia, July 2014

In this study, several reasons were identified as an indication for CS delivery. The most frequent indication was obstructed labor (30.7 %) followed by fetal distress (15.9 %), abnormal presentation (13.4 %), previous CS scar (7.9) and failure to progress (6.8) (Fig. 1). Of the total 222 women who had CS due to obstructed labor, 190 (85.6 %) women had obstructed labor on arrival and 34 (14.4 %) women had obstructed labor that happened within the hospital.

Obstructed labor was the leading obstetric indicator for CS among both referral cases and direct admissions (Table 3). All of the institutional maternal deaths and seven of the immediate newborn deaths were from referral cases.

**Table 3:** Comparisons of obstetrics indication of CS and mode of admission

Reasons for CS	Mode of admission	
	Referred cases	Direct admission
Obstructed labor	190(33.1)	32(21.5)
Feta distress/NRFHP	84(14.6)	31(20.8)
Abnormal presentation	80(13.9)	17(11.4)
Previous CS scar	40(7.0)	17(11.4)
Failure to progress	41(7.1)	8(5.4)
APH	34(5.9)	9(6.0)
Failed induction	21(3.7)	11(7.4)
Sever preeclampsia/eclampsia	18(3.1)	4(2.7)
Post date	15(2.6)	3(2.0)
Cord Prolapse	13(2.3)	3(2.0)
Multiple gestation	10(1.7)	3(2.0)
Failed instrumental delivery	10(1.7)	0(0.0)
Failed VBAC	7(1.2)	6(4.0)
Maternal preference	5(0.9)	3(2.0)
Prolonged PROM	6(1.0)	2(1.3)
Total	574(100)	149(100)

### Differentials of cesarean section delivery

After adjusting for other factors, residence (may be due to referral or selection bias), maternal age and presence of risk factor showed significant association with cesarean section. Likewise, the odds of undergoing cesarean section was 1.67 (AOR = 1.67, 95 % CI: 1.39, 199) and 2.31 (AOR = 2.31, 95 % CI: 1.74, 3.07) times higher among women from rural and having history of risk factors, respectively. Similarly women in the age category of 15–19 had 37 % lower (AOR = 0.63, 95 % CI: 0.43, 0.93) probability of CS delivery compared to age category of 20–34 years). Furthermore, the odds of experiencing cesarean section was 9.80 (AOR = 9.80, 95 % CI: 7.16, 13.42) higher if the woman had abnormal presentations. Similarly a women having history of previous cesarean section and fetal weight of 4000gm and more were 3.93 (AOR = 3.93, 95 % CI: 2.39,6.44) and 13.68 (AOR = 13.68, 95 % CI: 7.87, 23.78) times more likely to give birth by cesarean section (Table 4).

**Table 4:** Bivariate and multivariate analysis of factors associated with cesarean section delivery at Felegehiwot referral hospital, Bahir Dar, Ethiopia, 2013

Variables	Route of delivery		COR (95 % CI)	AOR (95 % CI)
	CS (%)	All vaginal (%)		
Model 1 (distal factors)				
Residence				
Urban	356(49.2)	1352(60.2)	1	1
Rural	367(49.8)	892(39.8)	1.69(1.44,2.00)	1.67(1.39,1.99)^*^
Maternal age				
15–19	35(4.8)	170(7.6)	0.61(0.42,0.88)	0.63(0.43,0.93)^*^
20–34	594(82.2)	1834(81.7)	1	1
35–49	94(13.0)	240(10.7)	1.27(0.99,1.62)	1.05(0.78,1.41)
Gravida				
Primigravida	335(46.3)	1167(52.0)	0.77(0.65,0.92)	0.86(0.71,1.04)
2–4	284(39.3)	839(37.4)	1	1
5 or more	104(14.4)	238(10.6)	1.28(.99,1.66)	0.86(0.64,1.17)
Presence of risk factors				
Yes	101(14.0)	140(6.2)	2.49(1.91,3.25)	2.31(1.74,3.07)^*^
No	622(86.0)	2104(93.8)	1	1
ANC history				
Yes	566(78.3)	1737(77.4)	0.97(0.79,1.18)	0.86(0.67,1.17)
No or unknown	157(21.7)	507(22.6)	1	1
HIV status				
Yes	23(3.2)	104(4.6)	0.76(0.49,1.17)	0.69(0.44,1.08)
No	700(96.8)	2140(95.4)	1	1
Model 2 (proximal factors)				
Abnormal presentation				
Yes	161(22.3)	81(3.6)	7.30(5.51,9.68)	9.80(7.16,13.42)^*^
No	562(77.7)	2163(96.4)	1	1
Fetal weight (*n* = 2780)				
<2500 gm	84(11.6)	265(12.9)	1.01(0.78,1.31)	0.85(0.633,1.13)
2500-3999 gm	522(72.2)	1758(85.5)	1	1
4000 gm and more	37(5.2)	34(1.6)	3.42(2.13,5.50)	3.93(2.39,6.44)^*^
Previous CS				
Yes	70(79.1)	19(20.9)	12.27(7.35,20.49)	13.68(7.87,23.78)^*^
No	653(23.6)	2225(76.4)	1	1

^*^ *P* < 0.05

## Discussion

Cesarean Section is a life-saving procedure for both the mother and the baby. Delay in deciding for it may be detrimental for both. On the other hand, premature and wrong decision may increase the maternal and fetal morbidity and mortality. The purpose of this study was to determine the magnitude of CS delivery and to identify factors leading to CS in Felegehiwot referral hospital.

The proportion of women undergoing CS delivery in this study was 25.4 %. This finding is consistent with studies conducted in other parts of Ethiopia [20, 21]. This magnitude may be attributed to high number of referral cases. Therefore, the observed proportion cannot be used as reference data for the source population. However, the result is insightful for researchers and program personnel, for example, all maternal and the majority of immediate neonatal deaths were observed in relation to CS delivery. Presence of unforeseen complications, delay in making decisions or inadequate care might contribute to the observed result. Some previous studies [4, 6], corroborated that CS does not confer safety and quality of obstetric care and hence may not prove reduction in maternal and neonatal mortality and morbidity.

Consistent with other studies [2, 13, 21, 26], the most frequent indication of CS observed in our study was obstructed labor. This was mostly due to last moment reporting or transfer of women with obstructed labor to the reference hospital from the periphery. On the other hand, injudicious use of oxytocic drugs or unjustified induction with prostaglandins without prior assessment of risk factors like fetal size, presentation, stage of labor, position and pelvic adequacy might also contribute for the observed over diagnosis of obstructed labor and subsequent emergency CS.

Consistent with a study done southern Ethiopia [20], the second most frequent indication of CS observed in this study was fetal distress. Fetal distress was diagnosed among 115 fetuses. Although using retrospective facility data is often difficult to validate, 84 (73 %) fetuses were diagnosed to have non-reassuring fetal heart rate pattern. As none of the fetuses were monitored by continuous electronic fetal monitoring system, over diagnosis of fetal distress is expected. Precise interpretation of fetal heart tracing and use of fetal PH might be effective in reducing cesarean section rate. Otherwise, inaccurate diagnosis of fetal distress would lead to unjustified use of CS. In general, our findings confirm the need for accurate assessment and better understanding of the mechanism underlying non-reassuring fetal heart rate pattern.

In the current study, mothers who had previous CS were more likely to have CS delivery than their counterparts. Unless there is a clear, compelling and well-supported justification for CS, a carefully supervised and justified trial of labor is necessary. Trial of scar in singleton pregnancies can be given to reduce rate of repeated cesarean section as the risk of uterine rupture is low [2]. In this study, only one third [27] of the women who had previous CS were allowed to have trial of vaginal delivery and 19 (63.3 %) of them had successful vaginal delivery. This finding is consistent with other researches [27–30].

Consistent with previous studies [26], mothers living in rural area were 1.67 times more susceptible to have current CS delivery than their urban counterparts. This observation may be due to the tendency that rural women are less likely to attend ANC and to get prepared for attending skilled delivery service. Last moment reporting or transfer to the reference hospital is very high in this study. Such challenges in seeking obstetric interventions need to be evaluated in further studies.

Similarly, the chance of undergoing cesarean section would increase as age of the mother increases [31–33]. In this study, women’s in age group of 15–19 years were 0.63 times less likely to undertake cesarean section as compared to age group of 20–34 years. The effect of age in this study could be explained by the possibility of pregnancy complication increment by age [25, 34, 35].

Like studies reported in different areas [29, 33] mothers reported as having pregnancy risk factors like diabetes and hypertension were at higher odds of undergoing CS delivery in this study. Presence of abnormal presentations, big babies which cause Cephalo pelvic disproportion or malposition, are also consistently reported in other studies [26, 27, 33, 36].

Most of the maternal deaths would have been prevented if general anesthesia was legitimately used. Likewise, the use CS for a dead fetus and the inadequacy of VBAC trial necessitate further explorative study. All of the immediate newborn deaths were reportedly due to respiratory difficulty after birth and which could have been prevented if appropriate and timely care has been provided. Challenges and difficulties are enormous while organizing an operation to save the lives of mothers and neonates in a resource limited setting, including this hospital. This hospital is often overcrowded by referrals from rural districts and majority of patients are either pregnant women with ruptured uterus, obstructed labor and hemorrhage among others. The chronic shortages of anesthetic drugs, inadequate supply of blood for transfusion coupled with acute shortage of trained anesthetist personnel among others are the glaring gaps that demand immediate intervention.

### Limitation of the study

Researches based on secondary data suffer from incompleteness and unreliable information. Use of primary data from the clients would have helped exploring other factors such as obesity, literacy and socioeconomic status. Referral cases might overestimate the true magnitude of Cesarean Section. We did not also assess the quality obstetric care being provided. Therefore, the use of this information for comparison and decision-making should consider the inherent limitation of the study.

## Conclusion

Obstetric factors occurring around birth, including obstructed labor and fetal distress were the main reasons leading to Cesarean Section rather than background characteristics assumed to be a risk. The results imply that there is a need for timely and accurate screening of women during obstetric care and, decision to perform cesarean section should be based on clear, compelling and well-supported justifications. In addition, training of hospital staff, health officers, midwives and health extension workers in emergency obstetric care as well as neonatal resuscitation skills, and use of partograph for appropriate decision to undertake CS are critical. Finally, ensuring access to life saving drugs, supplies, and adequate blood for transfusion are necessary to reverse the current situation. Further research with robust methodology is needed to explore the quality of care being provided and to corroborate or refute the present findings.
